# Investigating Systemic Metabolic Effects of *Betula alba* Leaf Extract in Rats via Urinary Metabolomics

**DOI:** 10.3390/metabo15070471

**Published:** 2025-07-10

**Authors:** Gregorio Peron, Alina Yerkassymova, Gokhan Zengin, Stefano Dall’Acqua

**Affiliations:** 1 Department of Molecular and Translational Medicine, University of Brescia, 25121 Brescia, Italy; gregorio.peron@unibs.it; 2 Department of Pharmaceutical Sciences, University of Padova, 35131 Padova, Italy; ayerk358@gmail.com; 3 Department of Biology, Selçuk Üniversitesi, 42130 Konya, Turkey; gokhanzengin@selcuk.edu.tr

**Keywords:** *Betula alba*, natural diuretics, polyphenols, urine, metabolomics

## Abstract

Background/Objectives: Herbal extracts from *Betula alba* (birch) are traditionally used for their purported diuretic effects, but scientific evidence supporting these claims remains limited. In this pilot study, we evaluated the short-term effects of a standardized *B. alba* leaf extract in healthy adult rats using an untargeted urinary metabolomics approach based on UPLC-QTOF. Methods: Two doses, 25 or 50 mg/kg, of a standardized *B. alba* extract were orally administered to rats. The extract contains hyperoside (0.53%), quercetin glucuronide (0.36%), myricetin glucoside (0.32%), and chlorogenic acid (0.28%) as its main constituents. After 3 days of treatment, the 24 h urine output was measured. Results: While no statistically significant changes were observed in the 24 h urine volume or the urinary Na^+^ and K^+^ excretion, multivariate metabolomic analysis revealed treatment-induced alterations in the urinary metabolic profile. Notably, the levels of two glucocorticoids, i.e., corticosterone and 11-dehydrocorticosterone, were increased in treated animals, suggesting that the extract may influence corticosteroid metabolism or excretion, potentially impacting antidiuretic hormone signaling. Elevated bile-related compounds, including bile acids and bilin, and glucuronidated metabolites were also observed, indicating changes in bile acid metabolism, hepatic detoxification, and possibly gut microbiota activity. Conclusions: Although this study did not confirm a diuretic effect of *B. alba* extract, the observed metabolic shifts suggest broader systemic bioactivities that warrant further investigation. Overall, the results indicate that the approach based on urinary metabolomics may be valuable in uncovering the mechanisms of action and evaluating the bioactivity of herbal extracts with purported diuretic properties.

## 1. Introduction

Assessing the mechanisms underlying the biological activity of herbal preparations represents an area of research with significant translational potential. Botanical extracts are widely used both in traditional systems and in modern complementary medicine, yet their complex compositions and multifaceted effects often limit mechanistic understanding. Bridging this knowledge gap is essential not only for validating traditional uses but also for optimizing the safety, efficacy, and regulatory approval of herbal-based interventions.

*Betula* is the largest genus in the birch family. This comprises more than 100 species widely distributed across the Northern Hemisphere. These trees have been used in traditional medicine for centuries, particularly for their purported anti-inflammatory and antiseptic properties [1]. Various parts of the birch tree, including the bark, leaves, and buds have been traditionally used to treat conditions such as rheumatism and inflammatory disorders [2,3] and, more recently, urinary tract infections [4]. Birch buds have been employed as cholagogues, particularly in Eastern Europe and Russia [5,6], while the bark has gained attention for anti-inflammatory and anticancer properties. Some of these uses are supported by in vitro and in vivo studies [1].

Among its well-documented therapeutic effects, birch has also been recognized for its mild diuretic properties. According to the European Pharmacopoeia, *Betula pendula* Roth (silver birch; syn. *B. alba*) and *B. pubescens* Ehrh. leaves, as well as hybrids of both species, are considered mild diuretics that can be used to promote urine flow in cases of lower urinary tract infections and renal gravel [7]. For this reason, birch extracts have gained significant commercial value during recent years and are used as bioactive ingredients in many dietary supplements and botanicals. Different formulations on the market incorporate birch extracts, either as standalone products or in combination with other herbal diuretics such as dandelion (*Taraxacum officinale*) and goldenrod (*Solidago virgaurea*). These supplements are often marketed for their purported benefits in promoting kidney health, reducing water retention, and supporting detoxification [8]. A growing number of botanical blends, herbal teas, and liquid extracts containing birch are now available, reflecting increasing consumer interest in natural diuretics. A recently published clinical study on the bioavailability and efficacy of birch leaf infusion provided support for its diuretic potential. This study demonstrated that birch leaf extract, when consumed as a tea, not only promoted fluid excretion but also exhibited anti-inflammatory effects that could support its use in managing urinary tract health [4]. However, despite their popularity, few clinical studies have addressed the efficacy of birch products. The lack of large-scale studies and the complexity of birch’s pharmacological profile indicate a need for more focused research to confirm its diuretic efficacy and elucidate the underlying mechanisms of action. In this context, metabolomics offers a powerful systems-level approach to profile small-molecule changes induced by herbal treatments. By capturing endogenous metabolic responses in biofluids such as urine, metabolomics can provide hypothesis-generating data on the modulation of physiological processes.

Regarding the phytochemical composition of birch, the bark is known for the presence of triterpenoids of the ocotillol and dammarane types. Oleanane, lupane, and fernane families, such as betulin and betulinic acid are also present, these latter being the most characteristic constituents of this species. Phenolic compounds are mostly described in the aerial parts belonging to the classes of cyclic and acyclic diarylheptanoids, flavonoids (quercetin, myricetin, kaempferol, and their glycosidic derivatives), catechins, and lignans [1,9]. The composition of the essential oil from the inner bark of *Betula pendula* has also been described, and the main components found were trans α-bergamotene and α-santalene [10].

In this work, we used the traditionally claimed diuretic activity of *B. alba* leaf extract as a proof-of-concept model to evaluate the potential of untargeted urinary metabolomics in detecting systemic metabolic changes. A chemically characterized birch leaf extract was administered orally to healthy adult rats for 3 consecutive days. Following treatment, the 24 h urine output and electrolyte excretion (Na^+^ and K^+^) were measured to assess diuretic endpoints. In parallel, untargeted UPLC-QTOF-based metabolomic profiling was performed to identify subtle biochemical alterations that might reflect broader physiological effects beyond fluid balance. By integrating conventional and omics-based approaches, this exploratory study aims to demonstrate how metabolomics can reveal the previously unrecognized biological effects of herbal preparations and guide future mechanistic investigations into their modes of action.

## 2. Materials and Methods

### 2.1. Chemical Preparation and Characterization of Birch Extract

Birch leaves were obtained from a local seller, and the identity of the plant material was confirmed by the authors. The leaves were ground, and 300 g of material was extracted in a Soxhlet apparatus for 4 h using 60% ethanol as the solvent (1 L). The liquid extract was evaporated under vacuum, reducing its volume to 1/5. The obtained liquid was diluted with water (1:1), mixed with 15 g of maltodextrin, and dried under vacuum in a rotary evaporator. The final yield was 30% (*w*/*w*).

LC-DAD-MS^n^ was used for the analysis of birch extract. A liquid chromatograph (LC) (1260 Infinity, Agilent Technologies, Santa Clara, CA, USA) equipped with a diode array detector (DAD) was used. This was coupled with a ion trap mass spectrometer (MS) (MS-500, Varian, Palo Alto, CA, USA) equipped with an electrospray ion source operating in negative ion mode (ESI-). A Synergy RP-max (150 × 3 mm, 4 µm, Phenomenex, Torrance, CA, USA) was used as the stationary phase. As the mobile phase, a mixture of 1% formic acid in water (A) and acetonitrile (B) was used. The gradient was as follows: 0 min, 5% B; 30 min, 100% B; 32 min, 100% B; 34 min, 5% B. The flux was 0.3 mL/min, and the injection volume was 10 µL. Calibration curves prepared from solutions of chlorogenic acid (5–150 µg/mL), hyperoside (5–100 µg/mL), and myricetin (5–100 µg/mL) were used for the quantification of secondary metabolites (phenolic acids, flavonoid glucosides, and flavonoid aglycones, respectively). Because a commercially available reference compound for betuloside quantification was lacking, salidroside (5–100 µg/mL) was used instead; this compound shares the chromophore and is commercially available.

### 2.2. Treatment Protocol

All experiments involving animals were approved by the Ethical committee of the University of Padova (CEASA 49571). Male and female Sprague Dawley rats [weight 100 ± 4 g (male) and 97 ± 5 (female)] were caged in a temperature- and photoperiod-controlled room with diet and water ad libitum. The animals were randomly divided into control and treated groups (*n* = 6 animals per group). The treated groups received a single dose (25 mg/kg or 50 mg/kg) of *B. alba* extract for 3 days. For the oral administration, the extract was mixed with peanut butter, and 0.4 g of the mixture was administered to the animals. The control group received the same amount of peanut butter, lacking the *B. alba* extract. After the 3 days of treatment, the animals were moved into a metabolic cage for the collection of the 24 h urine output.

### 2.3. Urine Output and Excretion of Na^+^ and K^+^

The Na^+^ and K^+^ composition in urine was measured using atomic flame absorption. Urine samples (2 mL) were mineralized with a mineralizer (START D, Milestone srl, Bergamo, Italy), using nitric acid and H_2_O_2_. After mineralization, the samples were diluted with 10 mL of ultrapure water (Milli-Q, Millipore, Burlington, MA, USA) and centrifuged at 4500 rpm for 15 min. The determination of Na^+^ and K^+^ was performed using a Varian 55B flame atomic absorption equipment.

### 2.4. Metabolomic Analysis of Urine Samples

Samples were diluted with 5% formic acid (FA) in water and then centrifuged at 13,000 rpm for 30 min. The supernatant was collected and filtered through a 0.45 PTFE membrane. Analysis was performed by means of UPLC-QTOF MS^e^. An Acquity UPLC coupled to a mass spectrometer (Xevo G2 ESI-QTOF, Waters, Milford, MA, USA) operating in positive ion mode (ESI+) was used. Chromatography was performed on a Waters BEH C18 column (2.1 × 50 mm, 1.7 µm, Waters, Milford, MA, USA) kept at 40 °C as the stationary phase, and a mixture of 0.1% FA in water (A) and 0.1% FA in acetonitrile (B) was used as the mobile phase. The gradient started with 90% of A and in 11 min reached 90% of B. The chromatographic and MS data of the eluted metabolites were extracted from raw chromatograms and MS spectra using the MarkerLynx XS application of the MassLynx version 4.2 Software (Waters). The extraction parameters were the following: XIC window: 0.05 Da; peak width at 5% height: 6 sec; marker intensity threshold: 20,000 counts; mass window: 0.1; retention time window: 0.5; noise elimination level: 5. Isotopic peaks were excluded from the analysis. A list of the ion intensities of each peak detected was generated, using retention time and the *m*/*z* data pairs as the identifier for each ion. The resulting three-dimensional matrix was exported as a .csv file and contained arbitrarily assigned peak index (retention time-*m*/*z* pairs), sample name (observations), and ion intensity information (variables).

Tentative identification of metabolites was performed by calculating the potential molecular formulas from their isotopic patterns in MS, using the Elemental Composition tool (Waters). The formulas with the highest confidence of fitting (>80%) were then used to search for possible candidates in libraries such as PubChem and Chemspider. In case the molecular formula could not be calculated, possible candidates were searched by comparing their accurate *m*/*z* with the Human Metabolome Database (www.hmdb.ca). Finally, the identification of the molecular candidates was confirmed by evaluating their fragmentation patterns in the MS^e^ spectra.

### 2.5. Statistical Analyses

UPLC-QTOF data were statistically analyzed using MetaboAnalyst 6.0 (www.metaboanalyst.ca) and the software R (version 4.3.2). Firstly, the preprocessed dataset was dimensionally reduced using the near-zero variance function (nzv) from the caret package of the R software. Then it was mean-centered and divided by the standard deviation of each variable (namely, autoscaling). The dataset was submitted to explorative unsupervised principal component analysis (PCA) to summarize the fingerprinting information content by means of a score plot. The dataset was then analyzed using classification models such as Random Forest and Partial Least-Square Discriminant Analysis (PLS-DA), with the aim of retrieving the metabolic features that characterize each study group. A 10-fold cross-validation was performed to test the robustness and predictive ability of the supervised model. A Volcano plot was used to identify the variables that mostly contribute to the differentiation of the control and treated groups. The statistical significance of these variables was finally verified with a non-parametric ANOVA (Kruskal–Wallis; FDR-adjusted *p* ≤ 0.05).

## 3. Results

### 3.1. Characterization of Birch Extract

**Figure 1:**
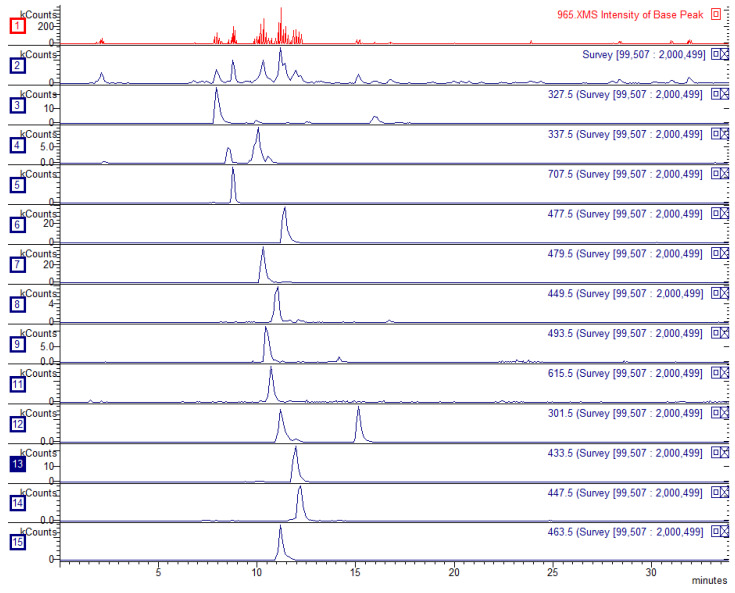
Extracted ion chromatograms of the most-representative compounds identified in *B. alba* leaf extract. Traces 1 and 2 show the base peak intensity chromatogram. Traces 3–15 show the peaks of betuloside, coumaroylquinic acid, chlorogenic acid, quercetin glucuronide, myricetin glucoside, myricetin arabinopyranoside, myricetin glucuronide, quercetin galloyl glucoside, quercetin pentoside, quercetin rhamnoside, and hyperoside, respectively.

The *B. alba* extract was analyzed by LC-DAD-MS^n^. Fourteen constituents were identified and quantified, corresponding to 2.73% *w*/*w* of the whole-extract composition. An exemplificative chromatogram showing the most relevant compounds with their extracted ion traces is reported in Figure 1. Overall, flavonoids such as hyperoside (0.53%), quercetin glucuronide (0.36%), and myricetin glucoside (0.32%) were the most representative compounds, along with the hydroxycinnamate chlorogenic acid (0.28%). All the results are reported in Table 1.

### 3.2. Urine Output and Excretion of Na^+^ and K^+^

The 24 h urine volume was measured in the treated (25 mg/kg dose and 50 mg/kg dose) and control groups. No difference between the two groups was observed in animals receiving 25 mg/kg of extract. For animals receiving 50 mg/kg of birch extract, the urine volume was increased by 15.4% compared with that in controls. Nevertheless, the observed variation was not significant (*p* > 0.05; 11.7 ± 2.9 mL vs. 13.5 ± 4.7 mL in control and treated groups, respectively). This may be influenced by the small number of treated animals and by interindividual differences in the urinary output.

**Figure 2:**
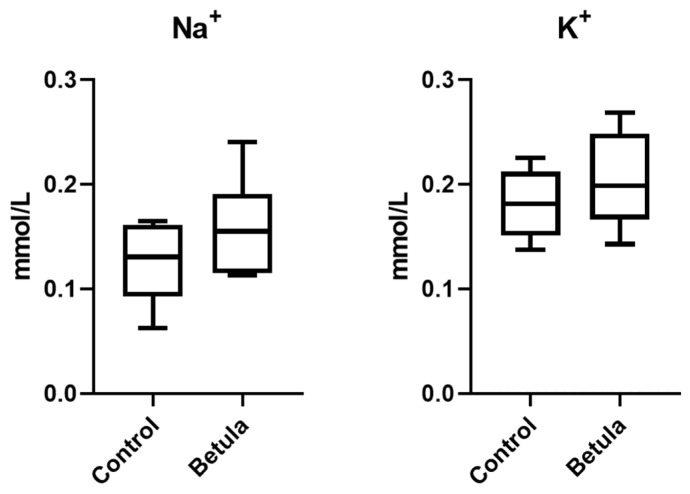
Concentration of Na^+^ and K^+^ in 24 h urine of rats treated with *B. alba* extract and controls.

Regarding 24 h Na^+^ and K^+^ excretion, the analysis was performed only on urine samples collected from the 50 mg/kg-treated group due to the negative results obtained with the lower dose. The results revealed a change after the birch extract administration (Figure 2). However, none of the variations was significant (*p* > 0.05): for Na^+^, the amounts in 24 h output of the control and treated groups were 0.13 ± 0.04 mmol/L and 0.16 ± 0.05 mmol/L respectively, while those for K^+^ were 0.18 ± 0.03 mmol/L and 0.20 ± 0.05 mmol/L.

Overall, the results indicate that treatment at the two doses did not exert a measurable effect in the amount of urinary output. Also, the treatment was not able to significantly change the sodium and potassium excretion or reabsorption in the kidney.

### 3.3. Metabolomic Analysis of Urinary Output

**Figure 3:**
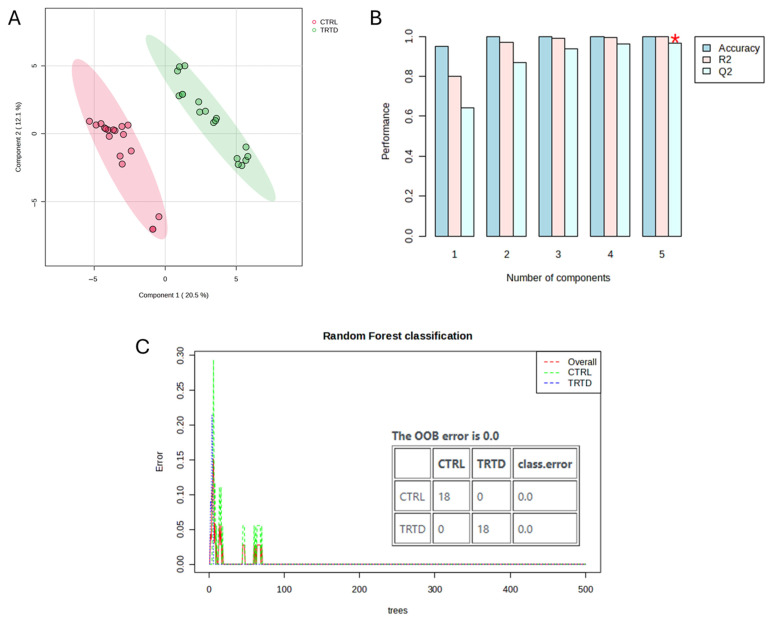
Results of the classification analysis of rat urine samples. Panel (**A**) shows the PLS-DA score plot where two distinct groups can be observed (red: control; green: treated with birch extract). Results of the 10-fold cross-validation of the model are reported in panel (**B**). R^2^ and Q^2^ values > 0.8 for the second component indicate robustness and good predictive ability of the model. Panel (**C**) shows the results from Random Forest analysis. Classification error rate was OOB = 0. * indicates the highest R2 and Q2 values, obtained for the component 5.

**Figure 4:**
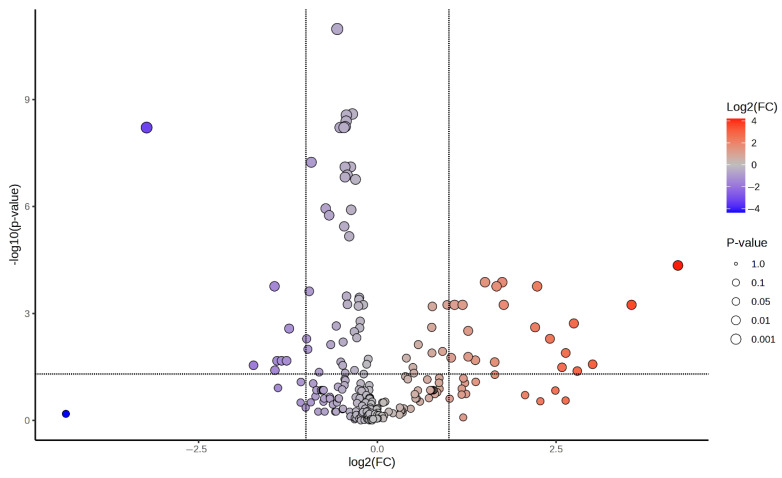
Volcano plot showing the variables associated with inter-group variability.

Untargeted UPLC-QTOF analysis of urinary output and data preprocessing allowed the collection of information on 741 features, defined by their accurate *m*/*z* values and retention time. A control vs. treated model was used to study the effects of treatment in the urine metabolome of adult rats. Specifically, the data were analyzed by performing a PLS-DA (Figure 3A,B), which successfully grouped the urine samples into two separate groups. The R^2^ and Q^2^ parameters resulting from the 10-fold cross-validation of the model were >0.8, thus indicating its robustness and good predictive ability. Classification of samples was also performed by Random Forest analysis, which revealed a robust classification of samples in “control” and “treated” groups (Figure 3C). The class error for the classification of the two groups was 0, as well as the OOB value. Variables outlining the inter-group variability were highlighted by a Volcano plot where the thresholds for variable selection were FDR-*p* < 0.05 and fold change > 2. As shown in Figure 4, 28 variables were highlighted: the amount in urine of 8 of these was decreased after treatment (log2(FDR) < 0), while that of 20 was increased (log2(FDR) > 0). These variables were tentatively identified by means of their MS and MS/MS parameters and are listed in Table 2.

Two glucocorticoids were tentatively identified among the variables increased in urine after treatment, namely corticosterone and 11-dehydrocorticosterone (11-DHC). A significant increase in 2,8-dihydroxyquinoline-beta-D-glucuronide was also observed, suggesting an alteration of gut microbiota activity due to treatment with birch extract, with the gut bacteria being involved in the formation of the precursor 2,8-dihydroxyquinoline from quinoline or 8-hydroquinoline [11]. A possible alteration of gut microbiota activity induced by treatment was indicated also by the increased excretion of bile acids, specifically glycocholic acid [12], 3-oxo-4,6-choladienoic acid, alpha-muricholic acid, and 7a,12a-dihydroxy-3-oxo-4-cholenoic acid. A variable, also induced by treatment, was tentatively identified as a cholic acid fragment (*m*/*z* 355.2634). In addition, an increased level of genistein 5-O-glucuronide was detected. This flavonoid may originate from a soy-derived ingredient that is present in the animal feed, with its enhanced urinary excretion possibly reflecting an upregulation of hepatic glucuronidation pathways following extract administration [13]. The overall pattern of elevated glucuronidated metabolites suggests a systemic response involving both liver phase II metabolism and gut microbiota activity.

Among the variables associated with the control group (i.e., decreased in urine after treatment) were two acyl carnitines (nonanoylcarnitine and deca-2,5,8-trienedioylcarnitine).

## 4. Discussion

Birch extracts are traditionally claimed to promote diuresis and support lower urinary tract health. Due to these purported effects, they are frequently incorporated as bioactive ingredients in food supplements. However, few studies have investigated their efficacy up to now, and their findings remain inconclusive [14]. For instance, no diuretic effect was observed after the oral administration of a mixture of dammarane triterpenoid esters of *B. pendula* to male Wistar rats [15], while two hours after the intragastric administration of one dose of 10 mg/kg extract of *B. pendula* leaves in rats, the urine output increased 1.3-fold in comparison with the control [16]. In our study, a *B. alba* leaf extract was administered to healthy adult rats, and its effects on diuresis were evaluated by measuring the 24 h urine volume and the urinary excretion of Na^+^ and K^+^. Monitoring the urine volume and electrolyte levels gives an indication of diuretic effects of a treatment, as changes in these parameters are indicative of alterations in renal function and fluid–electrolyte balance [17]. Sodium and potassium are key electrolytes that play crucial roles in regulating fluid balance, blood pressure, and cellular function. Diuretics often influence renal Na^+^ reabsorption, which in turn affects water retention or excretion. K^+^ excretion is also closely monitored because diuretics can alter its homeostasis [18]. However, the *B. alba* did not significantly alter urine volume or electrolyte excretion, suggesting it lacks conventional diuretic activity under the tested conditions.

To investigate the potential mechanisms underlying the extract’s biological activity, the 24 h urine samples underwent metabolomic analysis. The treatment induced significant changes in several metabolites, particularly steroid derivatives. Two glucocorticoids, i.e., corticosterone and 11-DHC, were tentatively identified as significantly elevated. Corticosterone is the primary glucocorticoid in rodents and plays a relevant role in the regulation of water and electrolyte balance [19]. Elevated corticosterone levels can influence fluid homeostasis by modulating hypothalamic–pituitary–adrenal axis activity and directly interfering with antidiuretic hormone (ADH) secretion. Glucocorticoids exert an inhibitory effect on ADH release through negative feedback mechanisms involving the hypothalamus and pituitary gland, potentially reducing renal water reabsorption by downregulating aquaporin-2 expression in the collecting ducts [20]. Additionally, corticosterone can impair the sensitivity of renal tubules to ADH, further diminishing water reabsorption efficiency. The observed increase in 11-DHC, the inactive oxidized form of corticosterone, may also be relevant. While traditionally viewed as an inactive metabolite [21], recent evidence suggests that 11-DHC may exert mineralocorticoid-like effects via conversion back to corticosterone in tissues expressing 11β-hydroxysteroid dehydrogenase type 1, particularly in the kidney. This metabolism influences the local corticosteroid milieu and may affect ADH-mediated signaling pathways and sodium retention [21]. However, the lack of significant changes in urine volume or electrolyte excretion observed in this study suggest that any suppression of ADH activity was insufficient to produce measurable diuresis, potentially due to counter-regulatory mechanisms or dose limitations.

In addition to corticosteroids, bile acid excretion increased in the treated group. Similar findings have been reported in animals treated with other natural products, such as brown algae [22] and gardenia [23] extracts. Recent studies highlight the role of bile acids in renal water regulation via the farnesoid X receptor (FXR) and the G protein-coupled bile acid receptor 1 (TGR5), both expressed in rodent and human kidneys. Deficiencies in FXR or TGR5 have been associated with reduced aquaporin-2 expression and impaired urine concentration capacity [24]. As bile acids act as agonists of these receptors and promote water reabsorption, their increase in the treated group is unlikely to account for any diuretic effect. Instead, these bile acids may be unrelated to urinary output and reflect other bioactivities, potentially at the intestinal level [25]. Notably, birch buds have been traditionally used in Eastern Europe and Russia as cholagogues [5,6], a function typically linked to decreased systemic and urinary bile acid levels due to enhanced biliary drainage and intestinal reabsorption [26]. In contrast, our findings suggest that the *B. alba* leaf extract exerts the opposite effect, as evidenced by the increase in urinary bile acid excretion following treatment. This observation implies a possible alteration in bile acid homeostasis, potentially due to hepatic modulation or impaired enterohepatic recirculation. Beyond digestion and renal signaling, bile acids are recognized for shaping the gut microbiota composition, affecting the balance between bile-metabolizing and bile-sensitive species [27]. Thus, the increase in urinary bile acids may indicate a disrupted intestinal bile acid pool, potentially triggering microbiota remodeling. This could influence systemic metabolism, as microbial metabolites like short-chain fatty acids, tryptophan catabolites, and secondary bile acids modulate host pathways via FXR, TGR5, and inflammatory responses [12,28]. In this context, the detection of 2,8-dihydroxyquinoline-β-D-glucuronide, a microbiota-derived metabolite, further supports the interaction of *B. alba* constituents with the gut–liver–kidney axis. These findings should be further investigated by integrating metabolomics and 16S rRNA-based microbiome profiling to clarify their mechanistic significance.

Treatment with *B. alba* extract was also associated with a reduction in the urinary excretion of bilin, a bile pigment. As elevated bilirubin levels and related pigments in urine are commonly correlated with hepatic dysfunction or hepatobiliary injury [29], the observed decrease may reflect a hepatoprotective effect induced by the extract. Nonetheless, while the metabolomic profile shows that *B. alba* leaves affect bile-related-compound metabolism and excretion, it remains unclear whether these effects are beneficial or potentially harmful. Overall, our data suggests that *B. alba* treatment modulates the urinary excretion of bile acids and pigments in rats, but further studies are needed to clarify the physiological implications for the liver and gastrointestinal health.

The treatment also induced the excretion of several glucuronidated metabolites, suggesting an effect of enhanced phase II biotransformation. Glucuronidation, catalyzed by UDP-glucuronosyltransferases in the liver, is a major detoxification pathway for both xenobiotics and endogenous compounds [30]. This pathway is frequently regulated by natural products. Our findings align with previous observations: a study reported increased glucuronidation in adult volunteers after consuming a *B. pendula* leaf infusion (3 g plant material in 150 mL boiling water) for 3 days [4]. Quercetin and kaempferol derivatives, likely transformed from extract constituents, were detected along with valerolactones from gut microbial polyphenol degradation. In our study, genistein 5-O-glucuronide, a flavonoid conjugate presumably from dietary sources [13], was detected. Additionally, two endogenous metabolites, i.e., hydroxy-methoxyindole glucuronide and 2,8-dihydroxyquinoline β-D-glucuronide, were elevated following treatment. These compounds likely derive from tryptophan metabolism. The former has been previously linked to detoxification under dietary restriction in mice [31], while the latter may derive from microbial or host metabolism of quinoline derivatives [28]. The same metabolite has been already described in rats in a previous study by our group, where a supplementation with cranberry induced its exertion with urine [13]. Although its physiological role is not fully understood, it has been proposed as a biomarker of peroxisome proliferator-activated receptor alpha (PPARα) activation [28]. PPARα is a nuclear transcription factor that regulates lipid metabolism, including fatty acid uptake and β-oxidation, and mediates peroxisome proliferation in response to certain xenobiotics [32]. Its upregulation suggests *B. alba* may modulate lipid metabolism via PPARα-related pathways, a hypothesis requiring further investigation. In this context, it is notable that nonanoylcarnitine, a medium-chain acylcarnitine involved in mitochondrial fatty acid transport, was significantly decreased in the treated group. Nonanoylcarnitine reflects mitochondrial β-oxidation activity, and its urinary levels can vary under different physiological and pathological conditions [33]. We have previously observed similar reductions following *Curcuma longa* extract supplementation. As seen during fasting, such decreases may indicate enhanced mitochondrial fatty acid uptake and oxidation [34]. However, in the current study, this remains speculative due to the lack of supporting data such as changes in body weight or other lipid-metabolism markers, underscoring the need for additional validation.

Finally, we identified cytosine-5-carboxylic acid (5caC), a modified nucleobase formed during active DNA demethylation. This compound arises from the oxidation of 5-methylcytosine by ten–eleven translocation (TET) enzymes and plays a role as an intermediate in epigenetic remodeling [35]. The detection of 5caC in urine may reflect increased DNA turnover or demethylation activity, possibly as a response to systemic effects of the extract. Such epigenetic modifications could impact the expression of genes involved in metabolism, detoxification, or renal function. While specific studies on urinary 5caC are limited, the presence of related demethylation products such as 5-hydroxymethylcytosine (5hmC) and 5-methylcytosine (5mC) in urine has been documented, supporting the concept that cytosine modifications are excreted and may serve as biomarkers of epigenetic dynamics [36]. The physiological relevance of 5caC excretion in response to botanical interventions remains to be determined and should be addressed in future targeted studies.

## 5. Conclusions

In this study, we applied an untargeted urinary metabolomics approach to investigate the short-term biological effects of a chemically standardized *B. alba* leaf extract in healthy adult rats. Although conventional markers of diuretic activity, such as 24 h urine volume and electrolyte excretion, were not significantly affected, metabolomic profiling revealed distinct changes in urinary composition. The most notable included elevated levels of specific glucocorticoids, bile acids, and glucuronidated derivatives of both endogenous and microbial origin. These shifts suggest that *B. alba* extract may influence steroid hormone metabolism, bile acid pathways, phase II detoxification, and possibly gut–liver–kidney axis communication. Additional modulations in acylcarnitines and osmolytes point to potential effects on energy metabolism and mitochondrial function, although these remain hypothetical and need further investigation. While the extract did not exhibit classical diuretic effects under the tested conditions, the observed biochemical responses support systemic metabolic activity. These findings align with the traditional use of *B. alba* for detoxification and fluid balance but also indicate broader physiological effects beyond renal function.

This study has several limitations. The small sample size and the absence of a positive control limit the generalizability of the findings. Although MS/MS data supported metabolite annotation, full structural confirmation was not achieved for all features. The short treatment duration may have precluded the detection of cumulative or delayed responses, and the lack of serum or tissue data limits correlation with systemic effects. Future studies should include longer treatment periods, appropriate controls, and multi-compartment analyses (e.g., serum, liver, kidney) to validate the systemic relevance of the observed urinary metabolomic changes. Further confirmation of the implicated pathways, particularly those related to steroid metabolism and hepatic detoxification, will also be essential.

Overall, this pilot study highlights the value of untargeted metabolomics in uncovering subtle metabolic responses to botanical preparations. It supports combining metabolomics with conventional pharmacological assessments and identifies *B. alba* as a promising candidate for further research into systemic metabolic effects and potential phytotherapeutic applications. To aid translation into human studies, relevant clinical endpoints should include liver function biomarkers (e.g., ALT, AST, bilirubin), adrenal hormone levels, and urinary metabolomic profiles. Changes in bile acid excretion, phase II detoxification markers, and renal concentrating ability (e.g., urine osmolality) may also serve as useful indicators of systemic activity in clinical settings.

## Figures and Tables

**Table 1:** Phenolic compounds identified in birch extract by LC-DAD-MS. Retention time (RT, min), *m*/*z* values of [M−H]^−^ ions, and *w*/*w* % of each identified metabolite are shown.

RT (min)	Identification	[M−H]^−^	Fragments	*w*/*w* %
7.6	Betuloside	327	147 119	0.22
8.2	Coumaroylquinic acid	337	191 163 127	0.04
8.4	Chlorogenic acid	353	191	0.28
9.5	Coumaroylquinic acid isomer	337	163	0.11
9.7	Myricetin glucoside	479	316 271 243	0.32
9.9	Myricetin glucuronide	493	317 179 151	0.10
10.1	Quercetin galloyl glucoside	615	463 301 271 179	0.05
10.7	Myricetin-pentoside	449	316 271 179	0.07
10.8	Hyperoside	463	301 151 179	0.53
11.0	Quercetin-glucuronide	477	301 151 179	0.36
11.5	Quercetin-pentoside	433	301 151 179	0.21
11.8	Quercetin-rhamnoside	447	301	0.15
14.7	Quercetin	301	179 151 107	0.13
	Total polyphenols			2.73

**Table 2:** Discriminant variables for the difference in 24 h urine composition of rats treated with *B. alba* extract and controls. Variables were selected using a Volcano plot, reported in Figure 4.

RT (min)	*m*/*z*	log2(FC) *	FDR-*p*	Formula	Tentative Identification (Adduct Type)	Main Fragments
3.4	370.2215	−3.2262	<0.0001	C_16_H_31_NO_4_	Nonanoylcarnitine ([M+H+HCOONa]^+^)	243.1599 85.0262
1.1	156.0401	4.204	<0.0001	C_5_H_5_N_3_O_3_	Cytosine-5-carboxylic acid	138.0298
5.6	430.2962	1.7467	0.0001327	C_26_H_43_NO_6_	Glycocholic acid ([M+H−2H_2_O]^+^)	466.3172
5.6	466.3172	1.506	0.0001327	C_26_H_43_NO_6_	Glycocholic acid	412.2855 448.3066
5.6	448.3064	2.2355	0.00017282	C_26_H_43_NO_6_	Glycocholic acid ([M+H−H_2_O]^+^)	412.2854
5.6	412.2856	1.6676	0.00017282	C_24_H_34_O_3_	3-Oxo-4,6-choladienoic acid ([M+H+ACN]^+^)	371.2551
1.5	158.1166	−1.4373	0.00017282	C_8_H_15_NO_2_	Pipecolic acid betaine	128.0686
5.6	931.6222	3.555	0.00057128	C_26_H_43_NO_6_	Glycocholic acid ([2M+H]^+^)	466.3172
7	332.3315	1.7681	0.00057128	C_23_H_46_NO_2_	Oleoylcholine ([M+H−2H_2_O]^+^)	ND
6.2	817.5814	1.1926	0.00057128	C_24_H_40_O_5_	Alpha-muricholic acid ([2M+H]^+^)	ND
6.2	355.2634	1.0807	0.00057128	C_24_H_35_O_2_	Cholic acid fragment	ND
1.1	118.0858	2.7488	0.0018969	C_5_H_11_NO_2_	Betaine	ND
3.6	338.0892	2.2069	0.0024467	C_15_H_15_NO_8_	2,8-Dihydroxyquinoline-beta-D-glucuronide	162.0558 134.0598
4.1	211.1421	−1.2327	0.0026492	C_9_H_21_N_2_O_2_	Trimethyllysine	ND
4.1	461.1088	1.2708	0.0030694	C_22_H_22_O_12_	Unknown	353.0505
5.8	405.2627	2.4142	0.0051249	C_24_H_36_O_5_	7a,12a-Dihydroxy-3-oxo-4-cholenoic acid	387.2562 369.2414
3.6	675.1685	2.635	0.012798	C_15_H_15_NO_8_	2,8-Dihydroxyquinoline-beta-D-glucuronide ([2M+H]^+^)	338.0891
4.4	447.0927	1.2704	0.016324	C_21_H_18_O_11_	Genistein 5-O-glucuronide	271.0601
6.3	329.2110	1.0332	0.017429	C_21_H_30_O_4_	Corticosterone ([M+H−H_2_O]^+^)	ND
4.4	345.2061	1.3707	0.020438	C_21_H_28_O_4_	11-Dehydrocorticosterone	ND
5.9	281.1041	−1.339	0.02119	C_17_H_25_NO_6_	Deca-2,5,8-trienedioylcarnitine	85.0277
4.8	299.1292	−1.2686	0.02119	C_19_H_14_N_4_	Bilin	273.1166
1.9	244.116	−1.4018	0.021233	NA	Unknown	NA
1.7	268.1049	1.6417	0.023064	C_10_H_13_N_5_O_4_	Adenosine	136.0623
3.7	340.1041	3.0114	0.026528	C_15_H_18_NO_8_	Hydroxy-methoxyindole glucuronide	164.0703
5.6	929.5964	−1.7314	0.028311	C_49_H_89_N_2_O_10_PS	PC(LTE4/P-18:0)	ND
4.3	164.0711	2.583	0.032292	C_9_H_9_NO_2_	3-Methyldioxyindole	146.0593 118.0655
1.6	483.2411	−1.4317	0.039056	NA	Unknown	NA
3.7	164.0711	2.7965	0.041092	C_9_H_9_NO_2_	3-Methyldioxyindole isomer	146.0593 118.0655

* log2(FC): a negative value of fold change logarithm indicates that the amount of metabolites in urine decreased due to treatment. Conversely, a positive value indicates an increase. The extent of variation is proportional to this value. NA: not assigned; ND: not detected.

## Data Availability

The original contributions presented in this study are included in the article. Further inquiries can be directed to the corresponding author.
